# New neighborhood, old habits? Delivery preferences of residents in new development areas and their assessment of alternative parcel logistics concepts: a case study of Berlin

**DOI:** 10.1186/s41072-023-00138-9

**Published:** 2023-04-07

**Authors:** Johannes Gruber, Benjamin Heldt, Saskia Seidel

**Affiliations:** German Aerospace Center (DLR), Institute of Transport Research, Rudower Chaussee 7, 12489 Berlin, Germany

**Keywords:** Last-mile delivery, Delivery preferences, Logistics concepts, Urban logistics, New development areas, Parcel locker, Micro-depot, Online shopping, Empirical survey

## Abstract

Various alternative solutions for sustainable last-mile parcel deliveries have been piloted and partially put into operation in Europe in the past decade. However, these delivery concepts have mainly been considered in inner-city areas. There are a few examples of the application of these concepts in peripheral urban areas, where new housing is being built to accommodate high population pressure. However, it is unclear whether the delivery preferences of residents in new neighbourhoods differ from those of the population average. This research conducted a case study in the western outskirts of Berlin, examining two newly built neighbourhoods and one existing residential area. Results from three survey waves of residents (N = 645) show that conventional doorstep delivery is preferred by 80% of the respondents. Nonetheless, there is a high willingness to use alternative delivery options, and respondents see benefits in climate-friendly delivery methods. This research also examines the willingness to pay for alternative parcel logistics concepts, which seems to be too low at the moment (at around €1 per shipment) to compensate for the additional costs of an operational change. However, the results also show an increasing awareness of and preferences for new delivery concepts, thus providing practical implications for planners and logistics operators alike.

## Introduction

Urban logistics face the challenge of simultaneously managing the continuous growth of online retail, satisfying changing delivery preferences, and significantly reducing negative traffic-related externalities. In the past decade, various solutions for last-mile deliveries have been piloted and partially put into operation, including micro-depots (also referred to as micro hubs), low-emission vehicles, and parcel lockers. So far, the mentioned new delivery concepts have been tested mostly in inner cities, that is, areas that are characterized by a high density of inhabitants and commerce, and thus the concepts have a higher likelihood of economic viability. Concepts are also being discussed for areas in the wider perimeter around cities; in North America, the suburban area plays a defining role, which is why there are also adapted delivery strategies of the operators (Rodrigue and Behrends 2018). There are fewer examples of urban concepts being expanded from city core areas towards medium-density peripheral urban areas (Morganti et al. 2014).

However, areas on the outskirts of large European cities are receiving increasing attention as housing construction often occurs there. Such areas are often characterized by a lower population density; but in addition to single-family houses, also multi-family houses can be found there. In contrast to suburbanization in North America, Europe continues to experience greater centralization and concentration (Hesse and Siedentop 2018). Areas adjacent to denser urban cores are expanding to a lesser extent into exurban spaces than in North America (Taylor 2011).

New development areas provide an excellent but often neglected opportunity to implement new delivery concepts for two reasons, one related to the consumer’s perspective and the other to the planning perspective.

First, a majority of private trips start from the place of residence. For example, home is the origin of 75% of all passenger trips in Germany (Nobis and Kuhnimhof 2018a, b) and therefore plays a very important role in mobility decisions. When people move to a new location, they often reorganise their everyday life according to the new conditions, which creates a window of opportunity for behavioural change (Müggenburg 2017), such as adopting new options for parcel delivery.

Second, new development areas make it possible for municipalities to plan or build new logistics infrastructure at an early stage. However, logistics solutions are often neglected when developing integrated mobility concepts (Heldt et al. 2021a, b) for new residential areas and, more generally, during the planning process (Assmann et al. 2019). New housing areas are often characterized by a rather suburban setting with low population density, few options to buy goods, and easy accessibility only by car. At the same time, due to the scarcity of land and the general trend towards sustainable planning, new housing is often planned to be space-efficient, densely populated, and less reliant on cars. Accordingly, it can be assumed that the population in these areas generates a high demand for deliveries.

Overall, little is known about the behaviour of residents of new housing developments and the conclusions that planning and practice can draw from this behaviour to promote both sustainable and suitable delivery concepts in these areas. For instance, it is unclear whether these residents’ ordering behaviour differs from those of the population average or from long-time residents in existing properties and whether alternative delivery solutions are compatible with their preferences. Furthermore, there is limited knowledge of the new residents’ willingness to use (WTU) and willingness to pay (WTP) for alternative delivery options.

This research aims to address this gap by examining several residential neighbourhoods in Berlin, Germany, and the freight-related online-shopping behaviour of people shortly after they have moved. In the rest of the paper, the state of knowledge regarding delivery preferences and willingness-to-pay for alternative parcel logistics concepts is outlined first (Chapter "Literature review"). Then, the background of the Berlin case study is described, followed by the “Methodology”. After presenting the main results of the residents’ delivery preferences and their assessment of alternative parcel logistics concepts, that is the expected individual benefits, WTU, and WTP in Chapter "Results", implications regarding these delivery concepts are highlighted in Chapter "Implications". This contribution concludes with recommendations for policy-makers and practitioners (Chapter "Conclusions").

## Literature review

### Parcel delivery preferences

Increasing online sales and the resulting rise in shipped parcels add to the pressure to search for sustainable parcel delivery solutions. In Germany, the shipped parcel volume rose by 50% from 2000 to 2018, totalling 3.5 billion shipments in 2018. This number has kept growing since, and in 2021, more than 4.5 billion parcels were shipped to German addresses (BIEK 2022). With regard to the number of parcels received by private recipients (B2C and C2C), there is little variation between the 16 German states (including Berlin): 2.5 parcels per person per month on average, 2.4 in Berlin, and a spread between the federal states of 2.3–2.8 (BIEK 2021). However, at a small-scale level, there can be high variations due to different socio-demographic structures: in the central district of Berlin-Mitte, the monthly order volume is around 4 parcels, while in the neighbouring district of Moabit it is only around 2 parcels (ibid).

There are various logistical options for delivering parcels to the end customer, although there are only two ways of actually receiving them: direct delivery to the residence or delivery to a pick-up point. Pick-up points include, for example, parcel lockers. Despite being widespread, only 12% of global e-shoppers use parcel lockers as a delivery address (Postnord 2020): Home delivery is the undisputed favourite. In combination with growing e-commerce, the dominance of doorstep deliveries is likely to increase the proportion of incorrect (first) delivery attempts. Failed delivery attempts, largely due to the absence of the consignee, are a common challenge of parcel logistics (Gevaers et al. 2009; Morganti et al. 2014; Van Duin et al. 2015). While incorrect attempts do not necessarily lead to a significantly increased traffic volume, they cause more stops and especially longer stop times leading to higher fuel consumption and emissions (Heldt et al. 2018).

Four-fifths of Germans state that home or doorstep delivery is their preferred method of delivery (Federal Environment Agency 2019). In a 2016 whitepaper, DHL found that 77% of German respondents prefer to receive their parcels at home. The second most-preferred delivery option was DHL’s parcel locker system ‘Packstation’. However, in case of absence, 50% of the receivers still preferred delivery to a neighbour (DHL 2016).

Some research shows that the dominance of home delivery can be countered. Molin et al. (2022) used a stated choice experiment to determine Dutch respondents’ preferences between home delivery, parcel lockers, and parcel shops. They found that even a small surcharge for conventional home delivery decreases the preference for this option considerably, from 71 to 7%. Based on another stated choice experiment, Merkert et al. (2022) found that for parcel receivers in Australia, postal home delivery is still the preferred option compared to aerial drones and parcel lockers. However, direct deliveries become less attractive compared to parcel lockers when there is no safe place to deposit the parcel at the recipient’s residence. Iannaccone et al. (2021) also examine home delivery versus parcel locker delivery using a stated preference survey in Rome. They found a high propensity to adopt parcel lockers, with distance and accessibility being the main determinants for considering the parcel locker option.

In addition to preferring doorstep delivery, Germans are increasingly demanding speedy delivery (e.g., delivery of fresh groceries within 10 min) or time-window deliveries (Joerss et al. 2016; Magloff 2020). While nowadays, only 21% of parcel receivers use time-window options offered by logistics providers, 82% are considering doing so in the future (GS1 2019). Overall, these developments will lead to further fragmentation of delivery traffic flows.

### Willingness-to-pay for alternative parcel logistics concepts

Delivery costs are high, especially for last-mile delivery (Gevaers et al. 2011). Therefore, knowing the WTP for alternative delivery options is of significant interest to logistics service providers. However, alternative delivery concepts are associated with higher costs, and therefore the relatively low WTP on the part of the end customer is seen as one of the main obstacles to implementing the concepts (Brabänder 2020). Källgren (2022) found that customers’ WTP for sustainable e-commerce deliveries varies substantially between European countries: the countries with the highest WTP are Italy and Germany, with 35% and 32% of customers willing to pay more, respectively, while those with the lowest are Poland and Finland, with 16%. This is in line with another survey that found that 70% of Germans are content with the cheapest form of home delivery independent of shipping duration or environmental impact (Joerss et al. 2016). Hagen and Scheel-Kopeinig (2021) stated that 26% of Germans are willing to pay €1 or more for alternative parcel delivery (using a micro-depot). Prümm et al. (2018) also analysed WTP for different parcel delivery options, such as time-window or speedy delivery. They found that around 1/3 of the German population would be willing to pay a substantial €2.34 extra for an environmentally friendly delivery. Therefore, studies agree that a quarter to a third of Germans would pay more for alternative delivery parcels, while the rest overwhelmingly prefer doorstep delivery.

While these studies analyse WTP for deliveries, none of them explicitly address the specific framework conditions of new housing developments or suburban residential areas. As stated in the introduction, doing so is interesting because these new areas provide opportunity spaces for behavioural change and new planning practices. The present study therefore aims to close this gap. In the following, the background of the Berlin case study is presented in the next chapter, followed by the research approach in Chapter "Methodology".

## Background of the case study

To present the background of this case study, an overview of the housing market in the German capital is given first (Section “Housing in Berlin”), followed by an introduction of the three study areas, which lie in the western edge of the city (Section “Study areas”), as well as existing logistics infrastructure (Section “Existing commercial transport and logistics facilities”). The section that follows (Section “Considered alternative parcel logistics concepts”) defines and describes three alternative parcel logistics concepts that were assessed by residents of the study areas. The final section (Section “Alternative parcel delivery options currently available”) shows which of these concepts are already available in the area today.

### Housing in Berlin

The city of Berlin has had strong population growth in the last decade. From 2011 to 2021, the number of inhabitants increased by around 10.5% to 3.68 million people (Rudnicka 2022). The medium population forecast expects further growth in the future: from 2018 to 2030, there will be a population growth of around 4.7%, equivalent to 177,000 people (Berlin 2020).

**Fig. 1:**
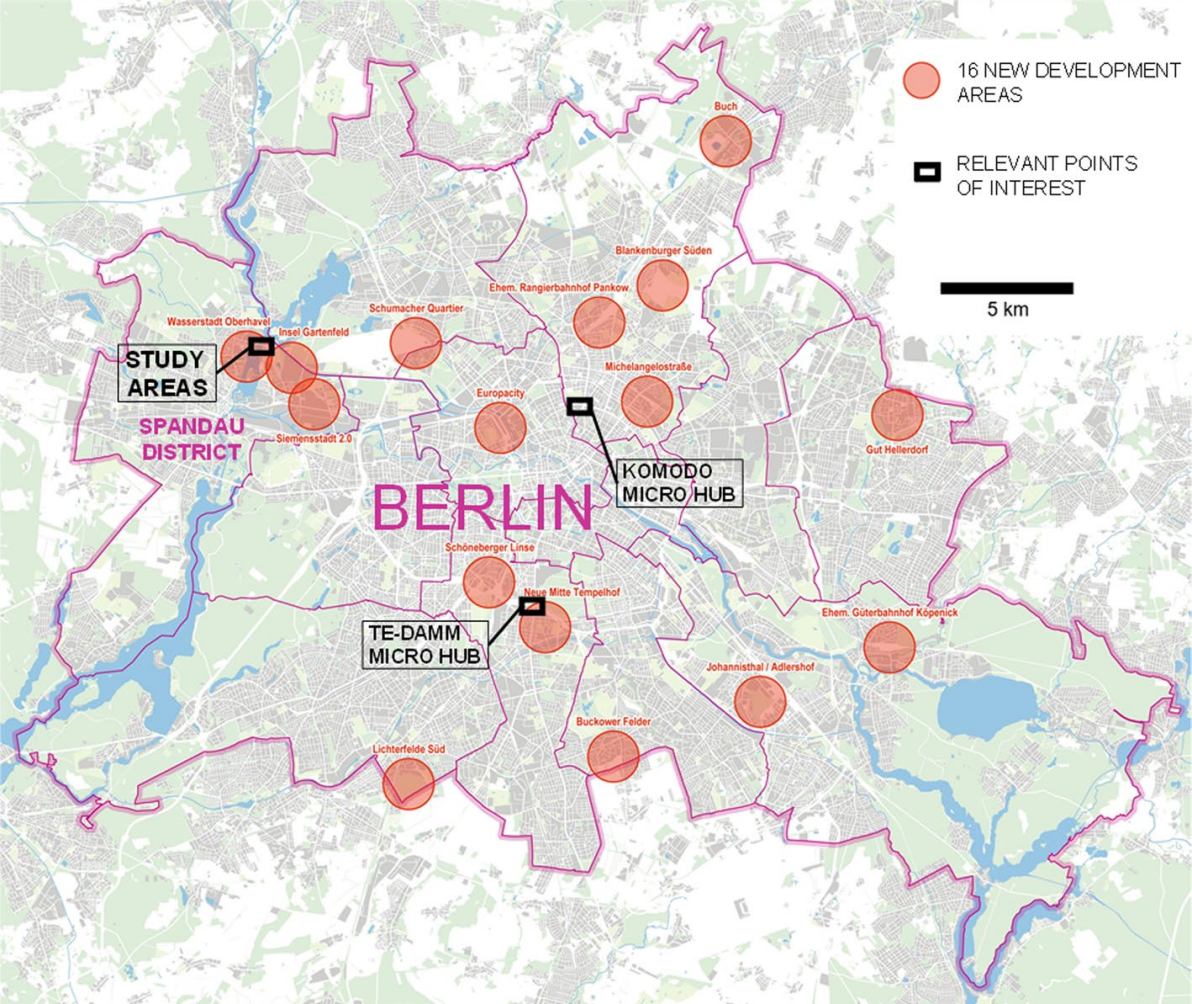
Map of new residential development areas and relevant points of interest in Berlin (based on Berlin 2019, modified)

Due to the strong growth of Berlin’s population, the Berlin state government has identified potential locations for the development of new housing. By 2021, it identified 16 areas as part of Berlin’s Action Programme to accelerate housing construction (see Fig. 1; Berlin 2021). Because of the low availability of space in the inner city, these new areas are often located on the outskirts of Berlin, which are characterized by a lower population density. Another consequence of the high pressure on the housing market is that urban logistics, housing, and other land uses compete for the available space in the city. Furthermore, people living on the outskirts are much more likely to use automobiles. The Berlin Senate thus strives to promote the development of car-reduced and more space-efficient neighbourhoods.

One important development area is the entire northeast of the district of Spandau, located in the west of the city. Two of the sixteen large new development areas, ‘Wasserstadt Oberhavel’ and ‘Insel Gartenfeld’, are located there (Fig. 1). Furthermore, Siemens will develop the Siemensstadt Innovation Campus in the area. The plan is to build around 22,300 residential units in the extended development area by 2030.

### Study areas

**Fig. 2:**
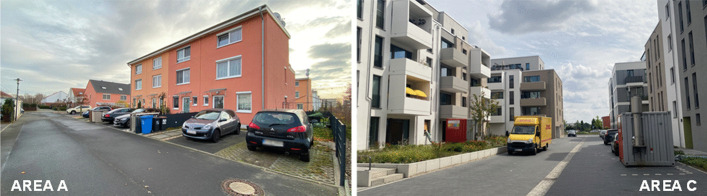
Pictures of residential buildings in study areas **A** and **C** (photographed by Heldt in 2021)

The questions posed in this article are examined using the example of three residential neighbourhoods within the area ‘Wasserstadt Oberhavel’ (Fig. 1). One residential neighbourhood has single-family houses (area A; see photograph in Fig. 2) and was planned by a private developer, while the other two have multi-family houses (area B and area C; see photograph in Fig. 2) and were recently developed by public housing associations; see Table 1. Before areas B and C were built, the study area was characterized by rather low-density housing (see area A) and a lack of stores. Currently, the distance to the nearest supermarket is more than 1 km. The closest main public transport stop is more than 2 km away and thus not within walking distance. Recent developments plan to accommodate several thousand people in the area in the next few years. They lead to a transformation of this suburban setting that creates challenges, such as how this many people can access basic supplies without stores in the area. In addition, these areas are designed to house only 0.4–0.5 cars per dwelling unit, which is, for instance, significantly below the parking requirement in Munich (of 1.0; see Munich 2007). Therefore, these areas are car-reduced developments. Thus, analysing the logistics system in this area, including demand characteristics and services, is very important for understanding the solutions these kinds of new neighbourhoods should implement.

**Table 1:** Information on the three study areas

Area	German name	Type of area	Constructed in year	Population (June 2021)	People per hectare
A	Haveleck	Existing residential area with single-family homes and row houses	2001–2014	1230	160
B	Pepitahöfe	Newly developed residential area with apartment buildings	2016–2019	2484	424
C	WATERKANT Berlin	Newly developed residential area with apartment buildings	2018–2025 (part A finished in 2020)	731 (part A)	461

**Fig. 3:**
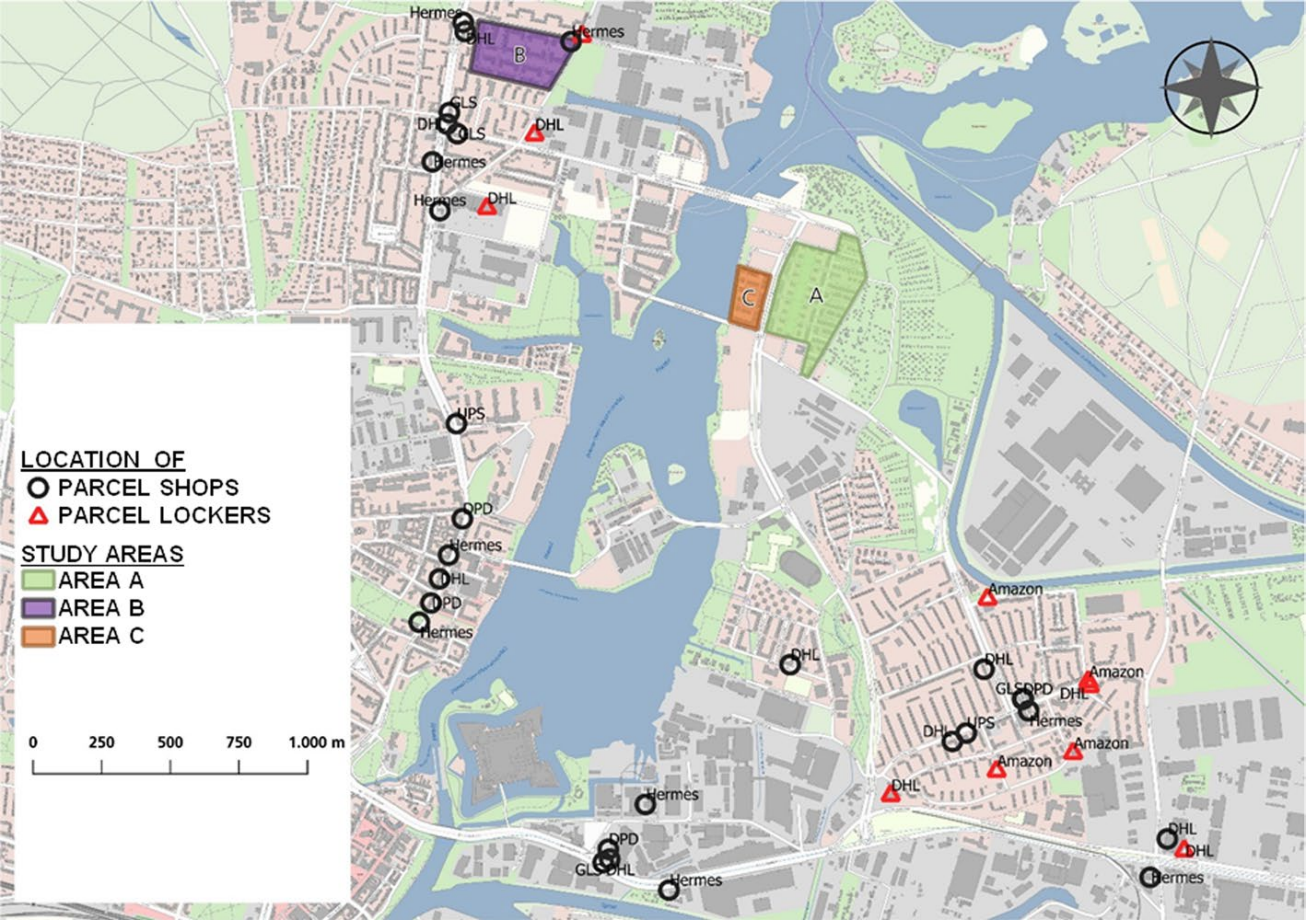
Location of parcel lockers and parcel shops in the wider vicinity of the study areas. Data source: Geoportal Berlin / WebAtlas Berlin, URL: https://fbinter.stadt-berlin.de/fb/wms/senstadt/k_webatlasberlin, Data licence Germany – attribution – Version 2.0, URL: www.govdata.de/dl-de/by-2-0

In the project ‘Move Urban’ (see acknowledgements), the mobility concept for the new car-reduced development area ‘WATERKANT Berlin’ (area C) was investigated, which combines several mobility solutions and aims to serve over 2,000 households in the near future (Heldt et al., in press). However, during the research project (2017–2021), only 360 dwellings were finished. The main component of the mobility concept is a mobility hub, which is currently located in the southern end of the area but will be located in the centre once construction is completed in 2025. This hub features station-based and free-floating carsharing and e-kick scooters and can also be used by residents from other areas, such as neighbourhood A. Thus, also area A was investigated, which has about 370 housing units. To understand the influence of a mobility concept, the main study area C was compared with the reference area B. The structure and location of area B are very similar to area C; however there is no mobility concept for area B. Area B comprises about 1,000 dwellings. Table 1, above, offers an overview of the three study areas (data sources: Statistics Office Berlin/Brandenburg 2021a/b). Areas B and C have a substantially higher population density than area A. Figure 3 shows a map with the location of the three study areas and the adjacent areas.

### Existing commercial transport and logistics facilities

Study areas B and C are currently under development. Accordingly, construction logistics play a dominant role. During the development phase, there are only few destinations in the immediate study area that attract commercial traffic. The existing commercial structure is limited to small and micro businesses. As the area is primarily residential, parcel delivery to private recipients constitutes the major share of commercial traffic.

There is no cluster of commercial parcel logistics locations (e.g. distribution centres) in the immediate locality of the study area. These tend to be located towards the city centre as well as along the city highway. Following a mapping of parcel distribution centres in Thaller (2018), it becomes evident that conventional parcel delivery with light commercial vehicles will have to drive 5–10 km each way to reach the destinations in the study area. Due to the proximity to distribution centers that continue to exist close to the city center, the trend of’logistics sprawl’ is less pronounced in the investigated area. Logistics sprawl refers to the relocation of logistics infrastructures from urban areas to the suburbs (Dablanc and Ross 2012), which has been observed worldwide for some time, for example in Chicago (Dubie et al. 2018), South Africa (Trent and Joubert 2022), but also outside of Berlin (Klauenberg et al. 2020).

### Considered alternative parcel logistics concepts

Due to increasing urban issues such as emissions, noise, congested streets, and double-parked delivery vehicles, alternative delivery concepts are being tested. These concepts consider new or alternative fuels or vehicles, bundling through collection points such as parcel stations, or a new organization of the logistics network. An expert survey (see Chapter 4) showed that practitioners from last-mile logistics and planning in Germany found the following concepts relevant especially for new construction areas: parcel lockers, concierge services, or cargo bike logistics (Heldt et al. 2021a, b). In North America, according to Rodrigue and Behrends (2018), operators tend to follow a dual strategy: new delivery concepts in urban areas and classic vehicles in suburban areas. Since the study area is on the edge of Berlin, but not yet "suburban" in the U.S. sense, the article focuses more on whether urban logistics concepts can work in lower-density neighborhoods. The relevant parcel logistics concepts and their current prevalence in Germany are presented below.

#### Parcel locker

A parcel locker is an automated container in which recipients can pick up the parcels they order. Residents can choose the address of the parcel locker and use it as a central delivery address for any goods of suitable size, or delivery companies may use them if they do not find the recipient at home. After the parcel is delivered to the station, the addressed person usually carries out the collection. Some companies also offer parcel-sending services through these lockers. There are several subtypes of parcel lockers according to location networks and the access option for logistics service providers:

**Decentralised parcel lockers** These parcel lockers are connected to residential buildings. They can be located inside buildings, for example, next to letterboxes, or outside but close to the buildings. The parcel locker can be permanently assigned to individual residents or flexibly used by several recipients.

**Centrally located parcel lockers** The parcel locker is not physically connected to a house but is centrally located or easily accessible in the residential area. Central parcel lockers are the predominant locker type in Germany currently. Heijs (2021) mentions the concept of parcel lockers at public transport nodes in the Netherlands, which is a typical sub-form of a central parcel locker.

**Proprietary parcel locker** Some parcel logistics providers have set up their own network of parcel lockers. These provider-linked parcel lockers are usually placed on the private property of landowners, supermarkets, or petrol stations. In particular, DHL (“Packstation”) and Amazon (“Amazon Locker”) use this type of locker to cope with increasing parcel volumes. DHL leads the German market with the “Packstation” concept and has been pushing for an ever-faster expansion of its network of lockers, which began in 2003: it had 4100 stations at the end of 2019 and 8200 stations at the end of 2021, and its target is to have 15,000 stations by the end of 2023 (Tagesschau 2021). The number of Amazon Lockers is around 400 (Intomarkets 2020). Instead, Hermes, DPD, and UPS are pursuing the establishment of their own parcel lockers to a very limited extent but are increasingly relying on parcel shops as an alternative delivery option.

**Open-supplier parcel locker** Open-supplier, provider-neutral, and white-label parcel locker are terms for parcel lockers used by various competing parcel logistics providers. This option is provided by a private company or an operator commissioned by the public sector. The website of a German courier logistics association (BdKEP 2022) provides data on more than 20 providers of white-label parcel lockers and related services. The collective use of a parcel locker can, in principle, achieve non-discriminatory access for different logistics service providers while simultaneously reducing competition for (public) urban space. Due to their potentially positive effects, several projects piloting these locker types have already been carried out (e.g. CityLog 2011). However, economic viability remains a hurdle: In February 2022, it was announced that DPD and Hermes would terminate their joint venture for an open-supplier solution in Germany (“ParcelLock”) for economic reasons (Hassa 2022): According to DPD, user numbers and the area coverage achieved had fallen short of expectations.

#### Parcel concierge service and parcel shop

A **parcel concierge service** is a service that receives goods or mail consignments on behalf of the customer. Additionally, the concierge can also take care of returns and, in some cases, provide fitting rooms. The concierge service may be central, i.e. serving the entire area (neighbourhood concierge), or decentral, i.e. within (each) apartment building. Concierge services are common in many US apartment buildings and also in so-called apartment communities. The concierge in residential areas usually offers a range of services (e.g. arranging repair services, and so on.). Thus, the handling of parcels is usually included in the service but incidental.

Unlike the concierge service, which offers services but not goods, parcel shops are retail businesses that use parcel reception as a secondary income opportunity alongside their actual business. The shops act as distribution partners for the parcel logistics providers. In Germany, all relevant operators have begun to offer such services in recent years. In 2015, for example, DHL operated 12,000 parcel shops (in addition to the Deutsche Post post-offices); Hermes operated 14,000; DPD 6,000; GLS 5,000 (currently, 7,000, according to the company website); and UPS 3,000 (Hillebrand und Junk 2016). In the following years, Hermes, in particular, pursued a massive expansion of its parcel shop network, aiming for 20,000 shops by 2020 (Bertram 2018). Usually, the contracts between parcel logistics providers and their local shop partners (e.g. kiosks) contain a non-competition clause, ensuring that a parcel shop represents only one provider. However, there are exceptions: a cross-provider parcel shop exists in Hamburg’s shopping centre City-Centre Bergedorf, which allows people to collect parcels from DPD, GLS, and UPS (initially also Hermes; Schmidt 2018; CCB 2022).

#### Micro-depot in combination with cargo bike delivery

Micro-depots, micro-hubs, or urban consolidation centres are transhipment points where goods are transferred from large transport units to cargo bikes or other light, mostly emission-free delivery vehicles. Micro-depots can be containers, swap bodies, or small warehouses (Arndt 2018). In addition to reducing emissions, the use of smaller vehicles can also solve problems such as minimizing double parking or decreasing the parking cruising time of delivery vehicles. Cargo bikes are increasingly used by logistics companies and are becoming more competitive (Gruber et al. 2014, Gruber and Narayanan 2019).

Micro-depots are considered an important prerequisite for deliveries with cargo bikes and must be located in the vicinity of the delivery area. Almost all international examples of micro-depots in combination with cargo bike deliveries are located centrally; this is the case for micro-depots in London (Leonardi et al. 2012), Brussels (Verlinde et al. 2014), Italy (Nocerino et al. 2016), and the Netherlands (Moolenburgh et al. 2019).

The additional transhipment in the transport chain requires space in which a micro-depot can be built or temporarily installed, which requires financial and organisational resources. The availability of suitable land is the biggest barrier to using micro-depots (Assmann et al. 2019).

In Germany, several large companies in the parcel industry have already piloted the micro-depot logistics concept in combination with cargo bikes, such as UPS, which has four very centrally located sites in Hamburg, and DPD/GLS in Nuremberg (Lenz and Gruber 2021). In Prenzlauer Berg, a densely populated urban district of Berlin, the five leading German parcel logistics providers participated in the ‘KoMoDo' project in 2018 and 2019, using a shared space for individual micro-depot solutions serving a 3 km delivery radius (see Figs. 1 and 9). However, there was no cross-company consolidation of shipments. Nevertheless, this project was one of the first examples of competitors cooperating under the umbrella of a municipal operator (the Berlin port and warehouse company BEHALA; LNC 2019). Another Berlin-based micro-depot project ('Te-Damm ') started in 2020 and is located centrally, close to the former airport Tempelhof (Berlin 2022a, see Fig. 1). A special feature of this publicly funded project is that it expanded the concept of the micro-depot to include warehouse storage by local producers. The approach shows that existing concepts can also be combined with other functions. In addition to these examples of (semi-)public micro-depots, there are about 70 privately operated micro-depots in Berlin, the majority of which (about 60) are used by a single provider (Berlin 2022b).

### Alternative parcel delivery options currently available

To date, parcel lockers and parcel shops are the most important alternative parcel delivery options available to the residents of the area. Fig. 3 shows the location of parcel lockers provided by various companies (e.g. DHL Packstation, Amazon Locker) and of parcel shops (provided by, e.g., DHL, Hermes, DPD, GLS, and UPS, the latter calling them’access points’) in the extended area (as of the beginning of 2022). The mapping shows that there are almost three times more parcel shops than parcel lockers in a radius of about two kilometres around area C. The number of Hermes parcel shops is significantly higher than that of the other market participants, including the market leader, DHL. However, there are no parcel lockers or shops directly in area C, the adjacent area A, and the neighbouring area to the south. In contrast, there are concentrations of these lockers and shops in an existing residential area at the southeast of the mapped area and the west of the Havel river. The residents of area B thus have several alternative options for receiving parcels in their immediate vicinity. In contrast, residents of areas A and C must go about two to three kilometres to pick up a parcel.

## Methodology

This section describes the methods of the case study (Section “Methods”) and the structure of the sample (Section “Sample description”).

### Methods

Several methods were used to evaluate the eligibility of logistics concepts for new residential areas in this case study. A standardized expert survey was carried out aiming to collate the experiences of practitioners and thus also assess the potential and effects of space-efficient transport and urban development in Germany. The survey was conducted in May and June 2018 online among 200 experts from all over Germany working in the fields of public administration, housing companies, and planning offices (for further details, see Oehlert 2019; Heldt et al. 2021a, b).

Furthermore, a total of 645 residents were surveyed in three survey waves in 2019, 2020, and 2021 in the abovementioned areas A, B, and C. The questionnaires asked about household indicators and mobility behaviour as well as ordering behaviour and the use of different delivery options. In order to collect the residents’ assessment of alternative concepts, the respondents were also asked to state the individual benefit of new services on a scale from 1 (none) to 5 (high). These services include some solutions for parcel delivery as well as general mobility concepts such as e-scooter rentals. These questions enabled us to compare the residents’ perspectives on the usefulness of delivery concepts and other mobility services.

The survey also asked about the WTP for alternative delivery options (such as parcel lockers or concierge services) and for the continuation of conventional doorstep delivery. Doorstep delivery is currently free in Germany, but some logistics companies are considering charging a surcharge for it. Seven categories were used when assessing WTP. To calculate the resulting average cost per shipment, the mean value of the value range provided by respondents was used. Thus, the following values were set for the response categories: ‘nothing at all’ = €0.00, ‘under €1’ = €0.50, ‘€1 to under €2.50’ = €1.75, ‘€2.50 to under €5’ = €3.75, ‘€5 to under €7.50’ = €6.25, ‘€7.50 to under €10’ = €8.75, and ‘€10 and more’ = €10.00.

The survey results were compared with a study by the Federal Environment Agency on Germans' online shopping behaviour (Federal Environment Agency 2019).

The mapping of the locations of parcel stations and parcel shops (Fig. 3) is based on an internet search (as of March 2022) on the websites of the providers DHL, Amazon, Hermes, DPD, GLS, and UPS.

### Sample description

Between 45 and 205 people took part in the surveys across the different areas and survey years, which corresponds to a response rate of between 11 and 25%. In total, 645 responses were included. Results are specific to these areas and then can be compared to the district of Spandau and the city of Berlin (Heldt et al., in press). The population structure in the recently developed areas B and C is characterized by rather large and young households (as compared to the Berlin average), while area A – which was completed almost a decade ago – shows even larger households with older residents on average. Furthermore, all areas are characterized by an above-average proportion of households with high socio-economic status (a variable that considers household income and household structure). Area A accommodates an even higher share of these households than B or C, which have a number of households with low status (see Table 2, showing additional data for Berlin and Germany from MiD 2017, Nobis and Kuhnimhof 2018a on cars per household and Nobis and Kuhnimhof 2018b on socio-economic status). Two-thirds of respondents have a high-school degree (Abitur), and most of them work full- or part-time. The average household owns 0.9 cars in the newer areas B and C (Berlin 0.6), while one-quarter lives without a private automobile. In area A, however, households own 1.5 cars on average (see Table 2). Car ownership levels are generally higher and bicycle ownership levels are generally lower compared to the entire city of Berlin. Half of the respondents across all areas possess a monthly public transport pass. Finally, new residents seem to be open to new mobility offers. For example, in area C, one in three respondents is registered with a free-floating carsharing scheme.

**Table 2:** Case numbers of survey waves and selected economic attributes of respondents

	Cases 2019	Cases 2020	Cases 2021	Socio-economic status (data from 2021 survey)			Cars per household (data from 2021 survey)		
				Low (%)	Medium (%)	High (%)	0 (%)	1 (%)	2+ (%)
Area A	94	73	65	10	27	63	8	44	48
Area B	205	–	116	20	40	41	22	63	15
Area C	–	47	45	15	44	41	26	62	12
Berlin				23	52	25	51	43	6
Germany				22	47	30	22	53	25

## Results

This section presents the survey findings, first addressing residents' delivery preferences (Section "Delivery preferences and current online shopping behaviour"), then their benefit assessment with respect to alternative parcel logistics concepts (Section "Benefits assessment of and willingness to use alternative delivery options"), and finally their willingness to pay for them (Section "Willingness to pay for alternative delivery options").

### Delivery preferences and current online shopping behaviour

**Fig. 4:**
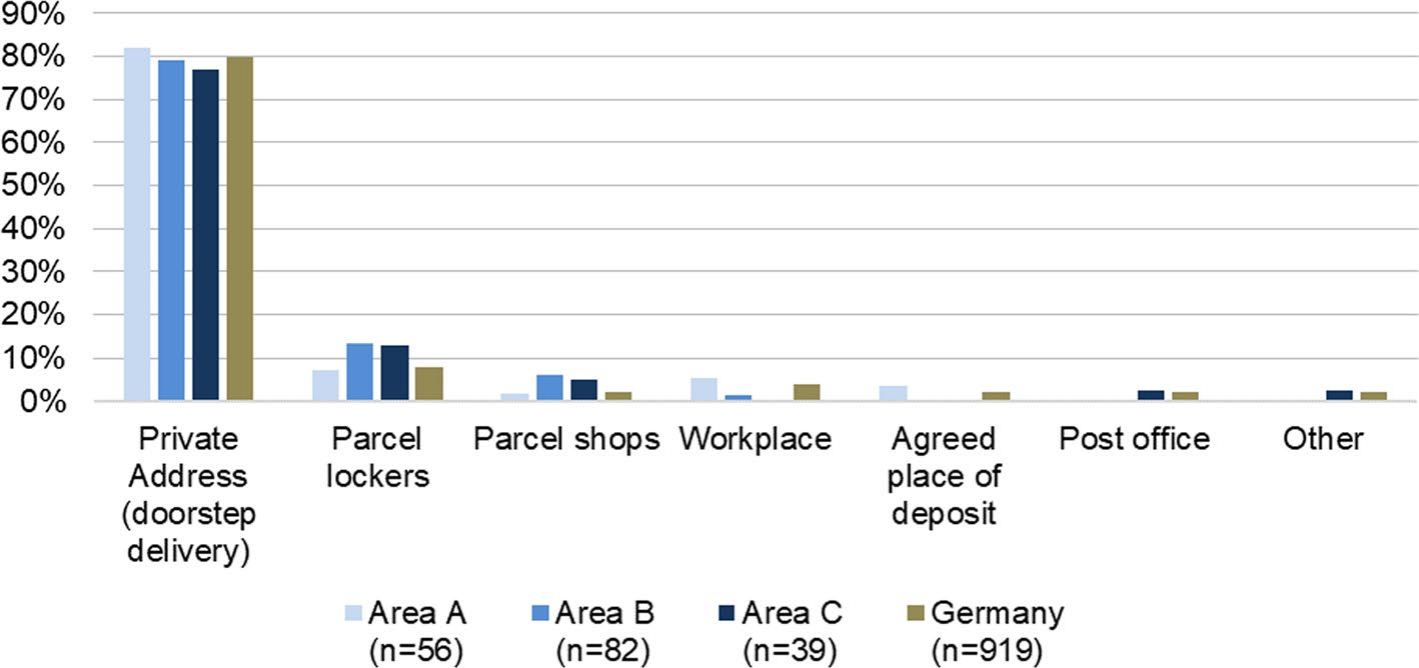
Comparison of preferred delivery locations

Fig. 4 shows the preferred delivery location for parcels (data from the third survey wave in 2021). In all areas, approximately four in five respondents prefer doorstep delivery, i.e., parcel delivery to their private address. This proportion can be compared with the study by the Federal Environment Agency (2019), which is representative of Germany. The self-determined values for the study areas are practically identical to the Germany-wide average. Thus, the standard preference is still largely parcel delivery to one’s private home address. Parcel lockers are currently the second-most popular form among the alternative delivery options, and, at 13%, they are more popular in the newly developed areas B and C than compared to the German average (8%) or area A (7%). A similar pattern applies to parcel shops, even though they are preferred by a much lower number of people: they are also more popular among respondents in areas B and C, yet they are the preferred option for only 1 in 20 respondents.

**Fig. 5:**
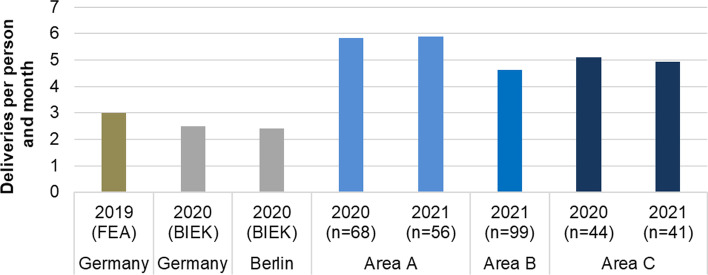
Parcel volumes of the respondents

While the difference in the preference for delivery locations between residents in new development areas and the national average is rather limited, the order volumes differ significantly. The representative German comparative value is three orders per person and month (Federal Environment Agency 2019, abbreviated FEA in Fig. 5). In contrast, in the study areas, the respondents ordered a significantly higher number of deliveries: between 4.6 and 5.9 deliveries per person and month (see Fig. 5). The highest values are in the already-existing row houses in area A. There is another opportunity for comparison with values for B2C/C2C parcel shipments stated by BIEK (2021), an association of parcel service providers. Data was collected differently than the values from FEA and this study and show lower values (despite the pandemic year 2020). Nevertheless, they also underline that the values determined for the three Berlin districts are significantly above the Berlin average.

Possible reasons to partially explain the significantly higher value for all study areas compared to Germany include the COVID effect in 2020/2021, which may have increased orders compared to 2019, the higher socio-economic status (see Table 2), and the internet affinity of respondents, and a possible sampling bias. It is plausible that residents of single-family homes in area A are more likely to order online as they experienced higher first-time delivery success at their private address than those in apartment buildings in areas B and C, where items are often dropped off at neighbours’ houses.

This study also queried the frequency of use of other types of delivery services. It found that restaurant delivery is the most frequently used service overall. In area C, around 60% of respondents ordered prepared dishes several times a month. The COVID-19 pandemic had a very clear impact on online shopping of fresh produce: before the pandemic, only one in ten used this service, while in 2021, this number doubled. Speedy deliveries, i.e., shipment within 90 min, show a similar pattern. These were not common in 2019 (e.g., only 2% of respondents in area A used them) and were used more frequently in 2021. Area C had the highest proportion of instant delivery users, at 15%. The effect of the pandemic on increasing the frequency of internet ordering is further supported by the finding that 70% of the respondents stated that they had replaced some physical food purchases with online buying in 2020.

### Benefits assessment of and willingness to use alternative delivery options

**Fig. 6:**
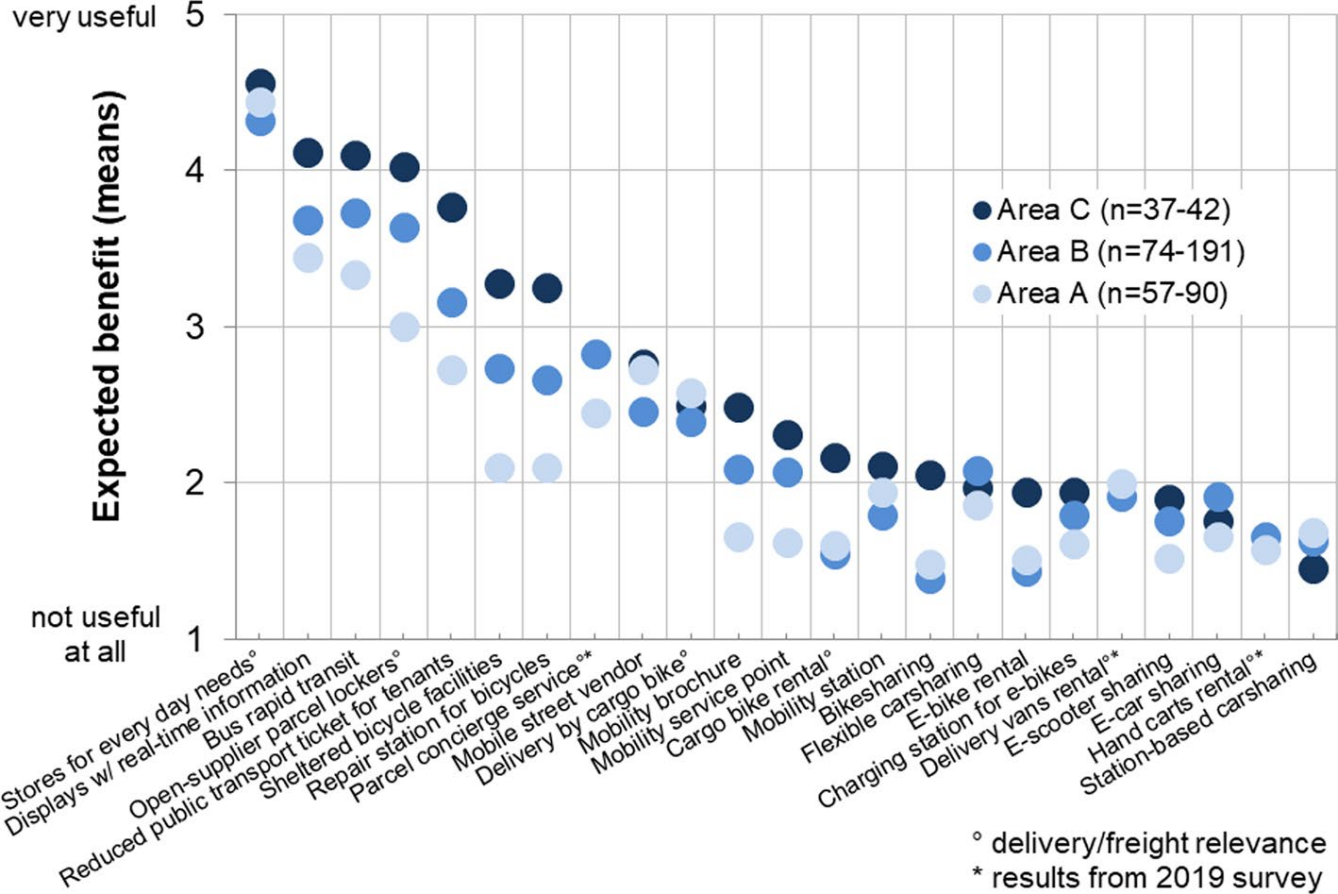
Survey respondents’ benefits assessment of a variety of mobility and delivery concepts

Residents in the three study areas were asked what benefits they would expect various mobility and logistics concepts to have. The study investigated more than 20 items (see Fig. 6). Items with direct or indirect relevance to freight transport are marked with the following symbol: °.

The first noticeable finding is that the surveyed residents in the new development area C expect a higher benefit from almost every mobility offer than the people in area A. The respondents expect that shops for daily needs will offer the highest benefit of all the offers surveyed. It is obvious that residents in new housing developments are keenly aware of the lack of such shops. For area C, the open-supplier parcel locker received a very high utility value of over 4.0 on a 5-point scale, being the fourth most-useful logistics concept surveyed. This value is similar for the other new development area B. One reason for this finding may be that the already available proprietary parcel lockers are currently the second most-popular delivery location in areas B and C (after doorstep delivery). Residents of area A, who live in row houses, assess the personal benefit of a parcel station as much more moderate at 3.0.

Respondents in all areas rate parcel concierge services and delivery by cargo bike, which requires the use of micro-depots, as similarly high with a moderate expected benefit of between 2.4 and 2.8. Those who receive parcels at their door generally do not perceive a difference between whether the parcel was transported by diesel van or cargo bike for the last mile. However, respondents assess the benefit of shifting towards cargo bikes as higher than zero, which means that some of the respondents view local emission-free parcel delivery as highly important. The residents view rental options that serve to transport goods as less relevant than other benefits. The offer that is seen as least beneficial is renting handcarts, while the rental of delivery vans or cargo bikes is slightly more relevant.

**Fig. 7:**
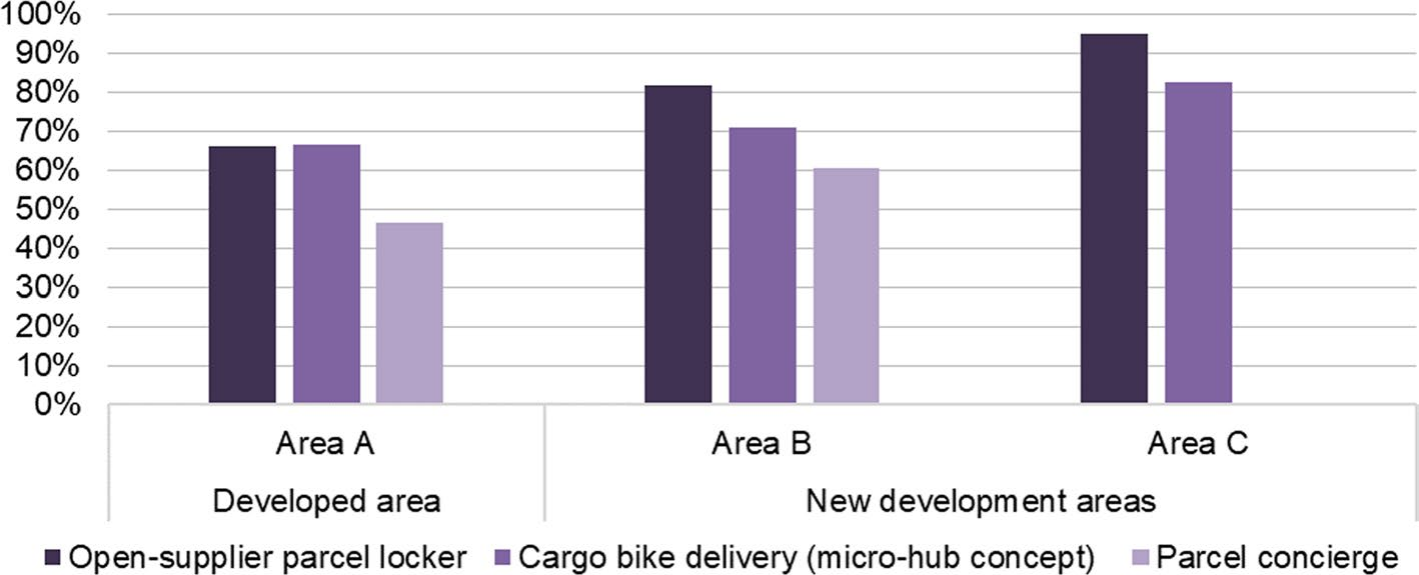
Willingness to use alternative delivery options

In the following, the basic willingness to use alternative delivery concepts is discussed (Fig. 7).

Overall, the propensity for using alternative delivery concepts is high. The respondents in the new development areas are more open to these concepts than the residents in the existing area A. Almost all respondents in area C (95%) would use an open-supplier parcel locker, while one-third in the existing area A cannot imagine doing so, and the value for area B is in between these figures. The WTU local emission-free delivery by bike (which requires transhipment at a micro-depot) is also quite high. The WTU a service in which goods are delivered by cargo bike is somewhat lower in the new development areas than the WTU an open-supplier parcel locker. Area A shows quite a similar value for WTU delivery by cargo bike: around two-thirds of the respondents said they would be open to it. Concerning parcel concierge services, 61% of the respondents in the new development area B and 47% in the existing area A would consider using such a service. This value is substantially lower than the values for the other concepts surveyed. The study did not survey the WTU parcel shops because this type of service is already available. As shown above, parcel shops are the third-most-popular delivery location in the study areas (see Fig. 4).

### Willingness to pay for alternative delivery options

**Fig. 8:**
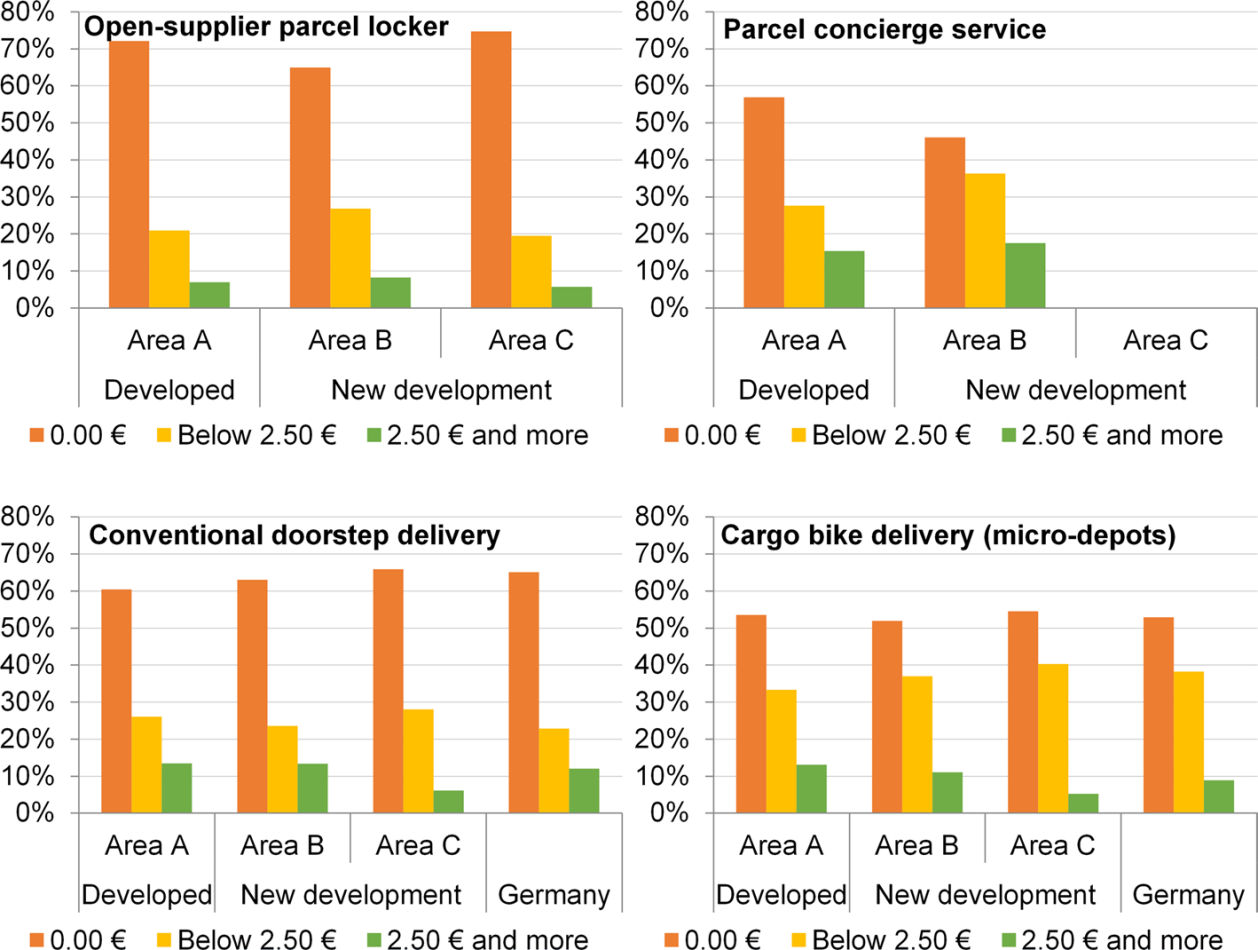
Willingness to pay for the considered parcel delivery concepts

The overall high WTU alternative delivery options is offset by a relatively low-to-moderate WTP for these options. Figure 8 shows the WTP for an open-supplier parcel station, a concierge service, conventional door-to-door delivery (with the hypothesis that additional costs would be incurred for this service), and an alternative doorstep delivery by cargo bike, i.e. a micro-depot concept, for areas A, B, and C.

The majority would not pay surcharges for any type of delivery. Three-quarters of the respondents in areas A and C would not pay any money for an open-supplier parcel locker. Area B deviates only minimally here. The WTP values for a parcel concierge service are higher, which may be because the survey participants are aware that the service is carried out by a human being. About one in six respondents in areas A and B would even spend €2.50 or more for this.

As discussed, for the (private) parcel recipient, a change from conventional doorstep delivery by diesel van to a micro-depot solution with delivery by cargo bike (also at the door) does not fundamentally change the delivery experience, if noticed at all. Therefore, the WTP for the concept ‘micro-depot & cargo bike’ is comparable with the hypothetical mark-ups for conventional doorstep delivery. In this case, as well, the majority would not be willing to pay more for either conventional or alternative forms of delivery. However, more people (around 45%-50%) would pay a premium for local emission-free doorstep delivery than for maintaining conventional delivery (around 35%-40%). This ‘extra’ WTP for climate-friendly doorstep delivery could be used economically by providers to offset the additional handling costs of shipping through micro-depots. However, when considering WTP, it must be taken into account that virtue signalling can also play a role when the use of environmentally friendly products is queried. Furthermore, socially desirable responses can distort the answers in empirical research. In reality, cost aspects are probably even more important decision factors and therefore the realizable WTP for environmentally friendly delivery alternatives may be somewhat lower.

Nevertheless, it is helpful to see the values for Berlin in relation to the German reference sample (see Fig. 8, bottom). The study areas do not deviate significantly from the average WTP. This applies both to a conceivable surcharge for conventional doorstep delivery and for delivery by cargo bike.

The WTP per shipment via different delivery concepts can only be roughly approximated as respondents were asked about a range of values (cf. chapter 4). Table 3 shows the assumed values. The respondents have the lowest additional WTP for a parcel locker, while the WTP a surcharge for a concierge service is approximately twice as high.

**Table 3:** Approximation of willingness to pay for surcharges per shipment

	Area A	Area B	Area C	All study areas	Germany
Open-supplier parcel locker	0.50 € (N = 201)	0.69 € (N = 268)	0.47 € (N = 87)	0.59 € (N = 556)	N/A
Parcel concierge service	1.00 € (N = 65)	1.28 € (N = 154)	N/A	1.20 € (N = 219)	N/A
Conventional doorstep delivery	0.96 € (N = 215)	0.95 € (N = 284)	0.61 € (N = 82)	0.90 € (N = 581)	0.69 € (N = 721)
Delivery by cargo bike through micro-depots	1.03 € (N = 183)	0.95 € (N = 235)	0.74 € (N = 77)	0.95 € (N = 495)	0.88 € (N = 879)

A look at the two variants of doorstep delivery reveals relatively similar WTP a surcharge, although delivery by cargo bike could generate slightly higher revenues than the hypothetical surcharge on the currently used conventional delivery concept. From the data presented here, the new development areas (B and C) do not generally have a higher WTP for sustainable delivery concepts than the previously developed neighbourhood A. However, the high economic status in area A (see Table 2) also plays a role in the overall higher WTP (with the exception of parcel lockers). Furthermore, residents in areas A and B have a higher WTP for conventional or alternative doorstep delivery compared to the German average, while the WTP of respondents in area C is lower than the nation-wide average, despite their higher socio-economic status than the national average.

## Implications

This chapter discusses the implications of the findings for the chances of success of the alternative parcel logistics concepts considered, namely for open-supplier parcel lockers (Sect. 6.1), parcel concierge services/parcel shops (Sect. 6.2), and micro depots/cargo bike logistics (Sect. 6.3).

### Open-supplier parcel lockers

The WTU open-supplier parcel lockers is very high. The proprietary solutions available are known and familiar, even though no parcel locker was available in the study areas at the time of the research. However, it is plausible that proprietary parcel lockers will be available for residents of the new development areas in the near future. The familiarity of these solutions, which are available throughout the city and can be used free of charge, is likely to contribute substantially to the fact that the WTP for an open-supplier solution is quite low.

Since the logistics service providers have little incentive to offer their services in cooperative systems, public actors such as the district of Spandau would have to get involved in establishing an open-supplier parcel station. Besides considering cooperating with a private operator, the district should also consider integrating a neutral municipal operator (similar to the KoMoDo project). A spatial connection to mobility stations or public transport hubs is desirable so that the location can be reached on foot or integrated into existing residential routes. This would also make the BVG (Berlin’s public transport company and operator of the existing mobility station on site) an important player in the possible expansion of open-supplier parcel stations in the city. However, in view of the low WTP, a further examination of the economic viability of this service is necessary, primarily for BVG, the district, the state of Berlin, and potential operators.

### Parcel concierge service and parcel shops

Although the stated WTP for parcel concierge services seems to be the highest for all the concepts surveyed, the concept is personnel- and cost-intensive and thus does not seem to be economically viable for the study area. Furthermore, private parcel shops are now available close to the new development area B (cf. mapping in Fig. 1). Such offers will probably also be created in the new development area C through cooperation between the parcel logistics services and the small-scale retail trade that is establishing itself in the area. Therefore, the public sector does not have to address this issue in these areas. Private sector involvement has created an alternative offer for parcel delivery in the form of parcel shops; a parcel concierge service is likely to remain reserved for luxury property locations.

### Micro-depots in combination with cargo bike delivery (in contrast to conventional doorstep delivery)

Our results indicate there is a WTP a surcharge for standard doorstep delivery and for alternative delivery by cargo bike (the WTP amounts to just under €1 per shipment). The similar WTP for both concepts implies that it is likely that parcel logistics providers will maintain the current process (standard doorstep delivery) and charge extra for it.

**Fig. 9:**
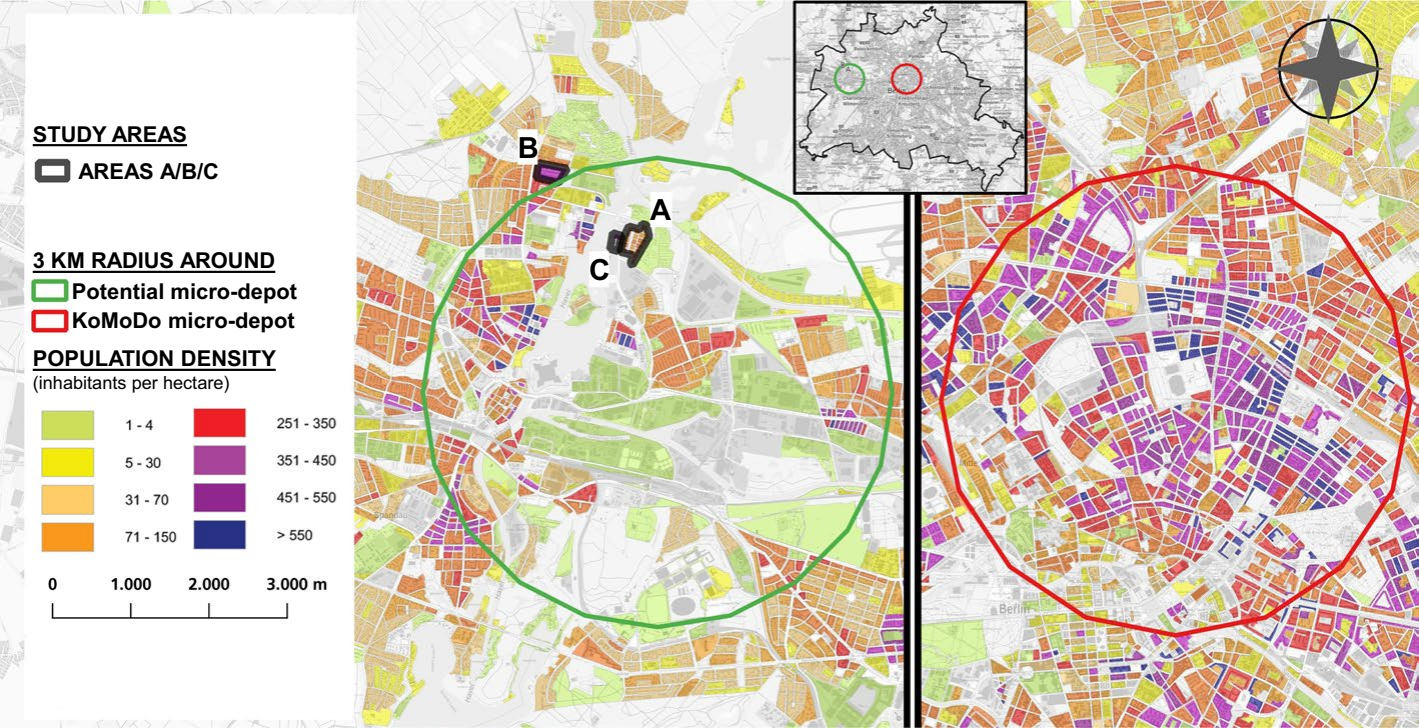
Population density in the study area (left) and the operational area of the KoMoDo micro-depot (right). Data source: Statistics Office Berlin/Brandenburg, licenced under CC-BY-3.0-Namensnennung, URL: https://creativecommons.org/licenses/by/3.0/de/

Should operators consider a change to cargo bikes, the costs for the additional space required by a micro-depot and for changing processes must be compensated by a sufficiently large volume of parcels in the area served. In the KoMoDo project, 160,000 parcels were delivered by cargo bike in 10 months (LNC 2019). Since the ideal service area for cargo bikes comprises a circle of up to 3 kms around the micro-depot location, there must be a high population density in the area. To visualise the issue with implementing this service, Fig. 9 shows the population density of a delivery area around a potential micro-depot location in the study area at an underground station (left) compared to the area served in the KoMoDo project (right).

As can be seen in the figure, aside from the differing population densities and urban structure, it is also evident that in the study area, delivery people would often have to travel 1–2 kms before they could reach the actual delivery area. In contrast to the inner-city area on the right, the traffic situation on the streets of the Spandau district is also more favourable for conventional vans and trucks. The findings show why a conversion towards micro-depot delivery currently only makes sense in areas with a higher population density and, thus, higher parcel volumes.

Furthermore, measures such as a change in parcel delivery method should not be enacted only at the neighbourhood level but at a higher level because of the scale of the logistical network planning and size of the delivery areas. Therefore, the City of Berlin should also support operators by considering logistics concepts in the higher-order planning processes, e.g. by developing a concept for the city-wide localization of micro-depots. This concept could then provide local authorities (e.g., districts and public housing associations) with guidelines for the provision of areas for micro-depots. Despite existing economic challenges, the implementation of these concepts can be tested in these newly built-up areas since infrastructure can be created more easily here. Thus, it is of crucial importance that instruments and guidelines ensure that low-impact delivery solutions are integrated into the planning of new housing areas from the beginning.

## Conclusions

This research offers several implications for planning practitioners and last-mile logistics operators willing to take advantage of the window of opportunity for freight-related behavioural change during residents’ relocation processes.

In general, the number of shipments in the study areas is significantly higher than the German average. This could raise the question whether car-reduced neighbourhoods may be associated with a higher number of deliveries. However, this research showed that in the comparison area where people have more cars on average the frequency of orders is similarly high. While some preferences of residents in new development areas do not differ significantly from the German average (such as the preference for home delivery), an above-average interest in alternative delivery concepts was found. Climate-neutral solutions are associated with a personal benefit which could be considered in the marketing or business model development of such solutions.

While the determined additional WTP for an economically friendly last-mile delivery is noticeably higher than for maintaining conventional doorstep delivery, the absolute WTP value of around €1 still seems too low for alternative business models in the suburban area to be economically viable without external financial support. Therefore, it can be assumed that the familiar business models already available in neighbouring locations—proprietary parcel lockers and parcel shops—will also become established in the new development areas and (initially) dominate the supply structure there.

Nonetheless, the results prove the importance of planning freight delivery in new development areas: above-average parcel order volumes are to be expected here, especially immediately after the move. It is crucial to include promising delivery solutions, such as freight transport concepts, in higher-level planning instruments and link them to transport development plans, urban development plans, and sustainable urban mobility/logistics plans (SUMPs/SULPs), so that the relevant stakeholders are given reliable framework conditions for their actions when planning new housing areas.

## Data Availability

The datasets used and analysed during the current study are available from the corresponding author on reasonable request.

## Abbreviations

BIEK: German Federal Association of Parcel and Express Logistics
FEA: German Federal Environment Agency
WTP: Willingness to pay
WTU: Willingness to use
